# Drug-Induced Epigenetic Alterations: A Set of Forensic Toxicological Fingerprints?—A Systematic Review

**DOI:** 10.3390/genes16101129

**Published:** 2025-09-25

**Authors:** Simone Grassi, Andrea Costantino, Alexandra Dimitrova, Emma Beatrice Croce, Francesca Iasi, Alessandra Puggioni, Francesco De Micco, Fabio Vaiano

**Affiliations:** 1 FT-LAB Forensic Toxicology Laboratory, Department of Health Science, University of Florence, 50121 Florence, Italy; simone.grassi@unifi.it (S.G.); emmabeatrice.croce@unifi.it (E.B.C.); fabio.vaiano@unifi.it (F.V.); 2 Forensic Medical Sciences, Department of Health Science, University of Florence, 50121 Florence, Italyfrancesca.iasi@unifi.it (F.I.); 3 Legal Medicine, Department of Prevention, ULSS 6 Euganea, 35131 Padua, Italy; alessandra.pugg@gmail.com; 4 Research Unit of Bioethics and Humanities, Department of Medicine and Surgery, Università Campus Bio-Medico di Roma, 00128 Rome, Italy; f.demicco@policlinicocampus.it

**Keywords:** DNA methylation, epigenetics, forensic toxicology, drug abuse, miRNA, histone modification, psychoactive substances, biomarkers

## Abstract

Background/Objectives: Epigenetics refers to heritable modifications in gene expression that do not involve changes to the DNA sequence. Among these, DNA methylation, histone modifications, and non-coding RNAs play a key role in regulating gene activity and are influenced by environmental factors, including exposure to psychoactive substances. In recent years, it has been hypothesized that such alterations may serve as molecular markers with forensic relevance. This systematic review aims to evaluate whether current evidence supports the use of drug-induced epigenetic changes as potential toxicological fingerprints in human subjects. Methods: A systematic literature search was conducted following PRISMA guidelines, including articles published on PubMed between 1 January, 2010, and 31 December, 2025. Only studies conducted on human samples and published in English were considered; animal studies and articles lacking epigenetic data were excluded. Results: Forty-two studies met the inclusion criteria. The most commonly investigated substances (alcohol, cocaine, methamphetamine, cannabis, and opioids) were found to induce specific and, in some cases, persistent epigenetic changes. These include alterations in CpG methylation in promoter regions, variations in miRNA expression, and modulation of epigenetic enzymes. Such changes were observed in brain tissue, blood cells, and semen, with evidence of persistence even after drug cessation. Conclusions: Current evidence confirms that psychoactive substance use is associated with specific epigenetic modifications. However, forensic application remains limited due to confounding factors such as age, co-exposures, and post-mortem interval. Further standardized research is necessary to validate their use as forensic biomarkers.

## 1. Introduction

Addiction represents a catastrophic event in an individual’s life [1]. It is defined as a chronic, relapsing neuropsychiatric disease that results from prolonged compulsion to use licit or illicit substances and continuous use in the presence of adverse medical, neurological, and/or psychiatric complications by vulnerable individuals [2]. This complex biopsychosocial disorder is related to the mesolimbic dopamine system, composed by the dopamine-producing neurons in the ventral tegmental area of the midbrain, connected to the medium spiny neurons, the main cell type in the nucleus accumbens (NAc) [3]. Both natural rewards and addictive substances rapidly boost dopamine signaling in the Nac which is strictly connected to the subcortical structure involved in reward-dependent learning, the corpus striatum [1,4]. As a response to chronic exposure to drugs, a long-lasting change in the structure, function, and gene expression of this brain region can be observed, associated with the persistent nature of addiction [5]. Recent studies showed that the susceptibility to addiction rises from environmental risk and genetic predisposition with a pivotal role mediated by epigenetic mechanisms [1,6]. Epigenetic mechanisms modulate gene expression by inducing chromatin compaction, which represses transcription, or relaxation, which facilitates transcriptional activation [7]. These regulatory processes are orchestrated by various epigenetic modifications, including direct changes to the DNA molecule (such as DNA methylation), post-translational modifications of chromatin-associated proteins (such as histone modifications), and the action of noncoding RNAs (ncRNA) involved in gene silencing [8]. Histones can be modified through the acetylation/deacetylation of the N-tails catalyzed by histone acetyltransferases (HATs, like Gcn5-related N-acetyltransferase or CBP/p300) and histone deacetylases (HDACs) [2]. DNA methylation is a covalent modification created by the addition of a methyl group (–CH3) to the carbon at position 5 of the pyrimidine ring of cytosine, mainly in “CpG” dinucleotides, by DNA methyltransferase enzymes (DNMTs) [8]. In the human genome, CpG dinucleotides are uncommon and often clustered in regions called “CpG islands”. Approximately 60–70% of human genes contain CpG islands in their promoter regions, which are usually unmethylated, and around 15–35% of all these islands are in promoter regions [9]. Hypermethylation of promoter regions is generally associated with transcriptional silencing, as it prevents the binding of transcription factors or engages specific proteins that promote chromatin condensation. In contrast, hypomethylation leads to greater accessibility and activation of transcription [8]. Remarkably, these epigenetic modifications can last even after the interruption of short-term substance use, potentially leading to long-lasting and even transgenerational effects [2]. There is a relatively significant amount of data about drug-related epigenetic changes in animal models, but very scarce evidence in human subjects/cells [10,11]. Moreover, despite research currently being focused on this evidence to improve the treatment of addictions, there is apparently no line of forensic research on the actual use of this information for forensic purposes.

Hence, the aim of this study is to verify whether there is enough evidence of this phenomenon on human samples to use it in the forensic context as markers of exposure to psychoactive substances.

## 2. Methods

**Figure 1:**
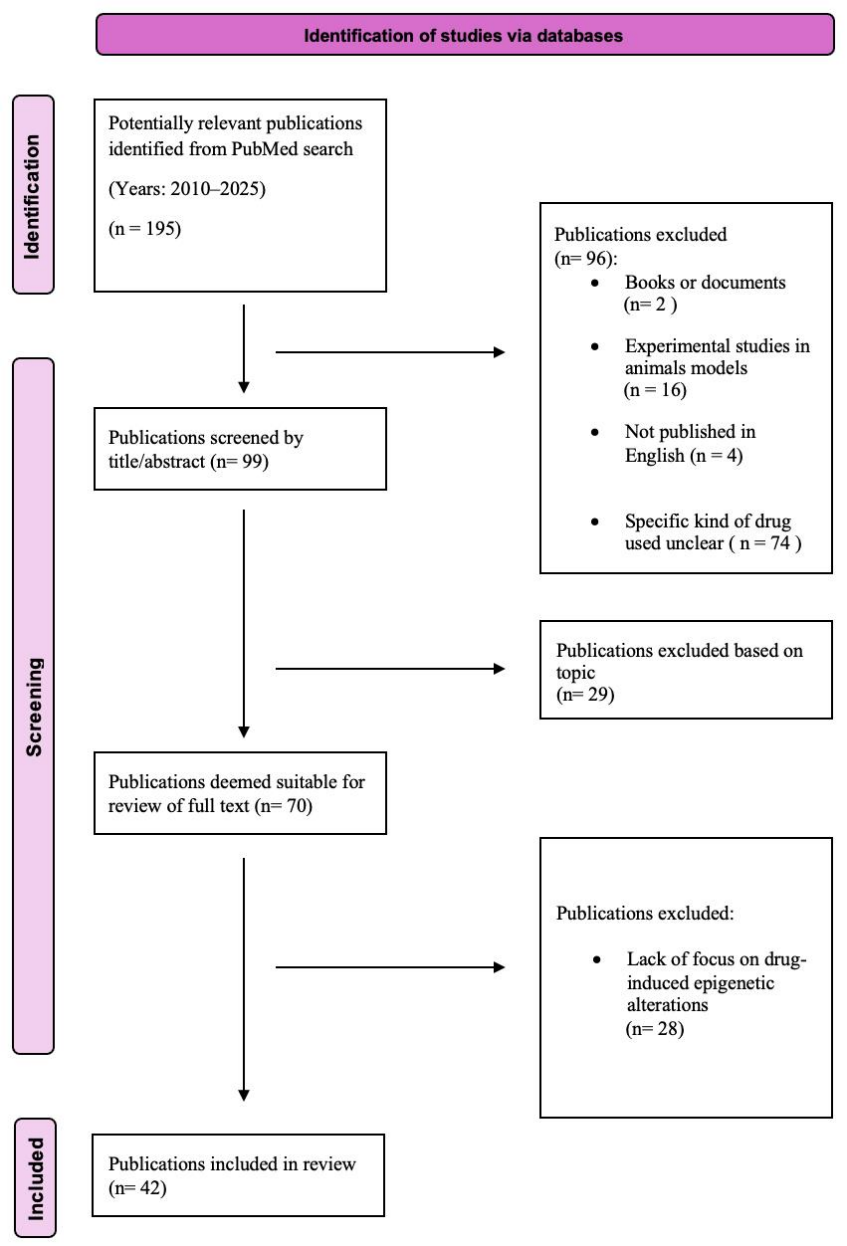
PRISMA flow diagram followed in this review.

We performed a systematic literature search according to the current Preferred Reporting Items for Systematic Reviews and Meta-Analyses Statement (PRISMA) criteria (Figure 1). We searched PubMed database for papers published between 1 January 2010, and 31 December 2025. We used a search string (restricted to the terms in the paper titles and abstracts) in which, using the Boolean operator “AND,” we combined the terms (“DNA methylation” OR “epigenetic*”) with the terms (“illicit substances*” OR “illicit drug*” OR “drug* of abuse” OR “substance* of abuse”). Our preliminary research identified 195 papers. A total of 96 papers were removed from the pool of eligible papers as they were books and documents, experimental studies in animal models, and articles that were not published in English. Of the 99 papers screened by title and abstract, 29 were excluded as they did not meet the inclusion criteria based on topic. Of the 70 articles remaining, 28 were excluded due to the lack of focus on drug-induced epigenetic alterations. Finally, a total of 42 eligible publications were included in our review and were critically reviewed by four investigators who extracted data relevant to the purpose of the present study. All authors agreed on the final data included in our study. A flow diagram of the articles selected for this review is summarized in Figure 1.

## 3. Results

### 3.1. Alcohol

In a large cohort (N = 8161, both males and females) 2504 CpGs associated with alcohol consumption were found, with the top 20 interesting genes substantially involved in brain/liver functions (SCL7A11, JDP2, GAS5, TRA2B, SLC43A1, PHGDH, PRPF8, ANKS3, TPD52L1, LAMA3, DHX16, DYRK2, SHMT2, SLC1A5, TCF3, and RNLS); 3 CpGs (cg10254445, cg15837522, cg18120259) remained unannotated [12]. Alcohol was also reported to increase methylation in PHOX2 A and in the promoter of NGF, but also to cause hypomethylation of GDAP1 and of DAT promoter CpG island 12 [13]. Moreover, in those who suffer from alcohol dependance (125 cases/69 controls, both sexes), a high methylation of three CpGs in the promoter of OPRM1 (that codifies for the μ-opioid receptor protein) was found [14]. An important marker is represented by DRD2 (dopamine receptor 2), whose hypermethylation is significantly positively correlated to the severity of the alcohol use disorder (AUDIT score: β = 1.139; t_648_ = 4.289; *p* = 2.07 × 10^−5^) [15]. Alcohol abuse can also alter methylation of the regulatory region of gene H19 and of genes DLK1 and GTL2 in the male gametes (40 men, alcohol consumers vs. controls) [16]. Focusing on histones and chromatin changes, alcohol abuse has also been associated with an upregulation of H3K4 histone methyltransferases and a global increase in H3K4me3 (an activation mark) in post-mortem samples of frontal cortex and amygdala [17]. Data in post-mortem tissues tend to be inconsistent, varying from global hypomethylation to sex-dependent extensive hypermethylation [18].

From a clinical point of view, inconstant hypermethylation of the DAT was also reported in the post-exposure withdrawal phase of alcohol use disorder, while methylation of MAOA and hypomethylation of NR2B and GDAP1 were observed in positive relationship to the severity of the alcohol use disorder [18]. On the other hand, hypomethylation of CpG of SCL7A11 (codifying for a cystine/glutamate transporter) was associated with an increase in heavy drinking days and liver function enzymes, including ALT and AST [12].

Alcohol intoxication has also been associated with upregulation of miR-34 in the human NAc, which are non-coding RNAs involved in addiction [19].

### 3.2. Cocaine

In the peripheral blood of those affected by cocaine use disorder (27 cocaine dependents/23 controls, both sexes), 186 CpGs of interest (61 hypermethylated and 125 hypomethylated) were found, related to 152 genes, mainly located in promoter regions [20].

Cocaine also decreases the expression of TET1 (codifying for enzymes involved in DNA demethylation) in the NAc [21]. In human hippocampus (25 cocaine users/25 controls, both sexes), chronic exposure to cocaine was related to changes in H3K4Me3 at gene promoters (the genome-wide changes were found to overlap between users of cocaine and alcohol, but cocaine-related changes were described as higher) [22]. In human primary astrocytes, a 24 h exposure to cocaine is able to decrease the expression of DMNT1 and DMNT3A (thus inducing global DNA hypomethylation) and to alter the levels of HDACs, decrease acetylation of HSK14 and HSK18, and increase the acetylation of H3K27 and H3K56 [23]. Regarding histone changes and chromatin remodeling, other authors reported that it upregulates the expression of HDAC1, HDAC4, and p300 and downregulates the expression of HDAC5 and GCN5 in human astrocytes [24]. Moreover, exposure of human neuroblastoma cells to cocaine causes temporary nucleosome repositioning in up to 223 genes (with number of genes proportional to the length of the exposure) [25].

Cocaine addiction has been associated with the downregulation of three miRNAs (miR-124-3p, miR-153, and miR-9), with one of them (miR-124-3p) being a fundamental regulator in cocaine-induced synaptic plasticity [1,19].

### 3.3. Methamphetamine

Use of methamphetamine was reported to lead to alterations in methylation of 235 CpGs, with a decreased methylation in the 54% of the loci (e.g., TTL7, SCN1A, and APBA1) and an increase in methylation of the remaining 46% (e.g., UNC5D, TGFBR3, and NET1) [26]. Moreover, methamphetamine exposure was reported to increase DNMT1 transcripts (in response to increased methylation level) in the human brain and hypermethylation of the Cry1 gene promoter region [13]. Epigenetic studies in specific pathological contexts have also been conducted. For instance, a statistically significant relationship was also reported on 171 patients (87 cases/84 control, both sexes) between methamphetamine psychosis and DNA hypomethylation of the promoter regions of DRD3, DRD4 and MB-COMT, involved in the catecholaminergic signaling and AKT1, encoding for serine/threonine kinase which regulates the neuronal growth/apoptosis [27]. Moreover, in subjects with HIV infection, methamphetamine use is associated with an increased global methylation of the frontal cortex, likely due to an increase ins the DNA methyl-transferase I [26].

Regarding histones and chromatin modification, methamphetamine was reported to upregulate the expression of HDAC1, HDAC4, and p300 and downregulate the expression of HDAC5 and GCN5 in human astrocytes [24].

miRNAs allow us to differentiate active methamphetamine users from recovering ones, since the former tend to show an increase in miR-4799, miR-4776, miR-550b, and miR-9, while recovering users exhibit a decrease in miR-181a, miR-15b, miR-let-7e, and miR-let-7d [19].

### 3.4. Cannabis

In the semen of 48 Cannabis users, 183 CpGs were found to be related to 177 genes and mostly (41%) intronic, with a significant correlation between the urinary drug amount and the level of methylation present [28]. In detail, 92 hypomethylated CpGs in a 62-nucleotide repeat sequence with 47.7 tandem copies are present within the aryl hydrocarbon receptor repressor (AHRR) gene [28]. Incubation of human peripheral blood mononuclear cells at a high THC concentration (200 ng/mL) was reported to downregulate DNA methyltransferases and upregulate ten-eleven translocation enzymes and receptors CB1 and CB2 mRNA levels [29]. Drug exposure has also been explored in specific pathological contexts, with cannabis chronic abuse in schizophrenic patients related to altered CpG methylation pattern at the CB1R promoter (in the peripheral blood lymphocytes).

### 3.5. Opioids

At the examination of the post-mortem orbitofrontal cortex of heroin users (15 heroin users/15 controls, both sexes), 1298 CpGs of interest (with hypermethylated regions enriched in exons and gene bodies and depleted in the promoters, while hypomethylated regions enriched in promoters and enhancers) were found [30]. Other authors reported that opioids (in particular, heroin) may cause hypermethylation of the promoter region CpG sites of OPRM-1 in white cells [13]. In human neuroblastoma cells, long-term (24 h) morphine exposure led to global DNA hypomethylation via altered EAAT3-mediated cysteine transport [31]. In particular, it caused hypomethylation of the CpG sites in LINE-1 (transposons) regions, while short-term (few hours) exposure induces an hypermethylation [31]. Regarding specific pathological contexts, in white blood cells of HIV-infected males, intravenous use of unspecified drugs (and concurrent HCV infection) was associated with differences in methylation of 6 CpGs in the promoter regions of four genes: a decrease in methylation in NLRC5 and TRIM69, and an hypermethylation in CX3CR1 and BCL9 [32]. Epigenetic alterations were found to last even after a 1 year from the last exposure to the drug [32]. Regarding histones and chromatin changes, they can upregulate the expression of HDAC1, HDAC4, and p300 and downregulate the expression of HDAC5 and GCN5 in human astrocytes [24]. Other authors reported that morphine application showed heterogeneous changes in HDAC expression in primary human astrocytes and increased acetylation of H3K9, H3K14, H3K18, and H3K27 [23].

Moreover, in optic nerve head astrocytes, a δ-opioid receptor agonist has been shown to increase the acetylation of histone H3, H2B, and H4 [33]. In post-mortem human brain (19 heroin users/17 controls), chronic heroin use causes histone H3 hyperacetylation and increased chromatin accessibility (particularly in glutamatergic genes), while acute intoxication leads to a repressed state of chromatin in the dorsal striatum [34].

Heroin abuse has also been associated with an increase in four long non-coding RNAs in the human NAc: MEG3, MIAT, NEAT1, and NEAT2 [35].

At the analysis of post-mortem Brodmann area 9 and peripheral blood (mean post-mortem interval: 26 h in 80 cases and 29 h 80 in controls), opioid use disorder was shown to lead in the dorsolateral prefrontal cortex to an increase of 29 and a decrease of 60 miRNAs, and in the blood to an increase in 51 and to a decrease in 53 miRNAs [36].

## 4. Discussion

Psychoactive substances not only can alter neural cells’ function, but may also cause oxidative stress and induce epigenetic modifications, that should be considered as adaptive responses [2,24]. We performed this systematic review to evaluate whether current knowledge about the epigenetic changes caused by drugs in human tissues is enough to allow a translation into forensic practice. Indeed, forensic applications of these kinds of markers could be many, for instance, to evaluate the fitness to drive in the driving license regranting process or to evaluate the history of abuse of psychoactive substances in criminal proceedings [37,38,39,40]. Epigenetic analysis is relatively complex and expensive, but hypothetically it could serve as confirmation test in ambiguous situations or when traditional toxicological methods are limited. For instance, our review showed that epigenetic changes are mainly consistent after long exposure to a specific drug, and in these cases the current gold standard is performing hair analysis. However, this analysis is limited, for example, when the person has short/no hair, when the exposure to the drug is relatively recent, or in the case of extreme hair treatments (i.e., blanching) [41]. So, in these cases, while traditional tests (urine or blood) can only detect recent drug use, in the absence of keratinic matrix, DNA methylation signatures in peripheral blood cells can show signs of past exposure to drugs for extended periods, potentially weeks to months after cessation [42]. Moreover, its sensitiveness is limited for very common substances of abuse like alcohol and cannabis. Other potential forensic applications of drug-induced epigenetic changes could be found in postmortem toxicology, and transgenerational and environmental investigations, for instance. When biological matrices are compromised by decomposition, epigenetic markers could offer an innovative way to detect signs of intoxication. After death, the relative stability of DNA methylation patterns in comparison to drug metabolites makes these markers potentially useful in cases involving long-term substance abuse history [43]. Another potentially interesting use of these epigenetic changes could be projected in every setting where transgenerational or environmental exposure to a substance is suspected. As seen, these changes can be transmitted to subsequent generations leading to increased susceptibility in offspring who were never directly exposed, opening new perspectives about parental liability. In order to highlight the potential forensic relevance of epigenetic biomarkers, we have added a comparative summary (Table 1) outlining their main advantages and limitations in relation to conventional toxicological methods routinely applied in forensic practice. Moreover, where habitat-specific epigenetic signatures are found in specific populations, these findings can be used to reveal exposure to particular chemical pollutants [44]. As shown, current epigenetic information is mainly obtained from animal models and is mainly targeted for clinical purposes. For instance, drug-induced epigenetic changes are being considered for therapeutic purposes: for instance, methamphetamine is able to increase the expression of CCR5 (a chemokine receptor) in myeloid cells and HIV load and, at the same time, inhibits HIV-1 replication in CD4+ T Cells through anti-HIV-1 miRNA [41,45]. Moreover, an interesting use of epigenetic information is also represented by the so-called “epigenetic clock” (i.e., use of epigenetic information as a marker of biological age): Huang et al. reported that illicit drugs (in particular, ketamine) may accelerate this clock in prefrontal cortex and NAc (involved in the drugs’ reward circuitry) [46].

We found that despite there being some forensic literature on applications like age estimation, the actual use of epigenetic analysis for practical forensic toxicology purposes has never been reported [47]. However, as shown by our review, a tentative set of markers could be hypothesized based on the most robust studies (see Table 2).

Non-codifying RNAs (in particular, miRNAs) should be considered particularly promising, because the human brain contains a very high variety of these molecules, since they concur in regulating its development and plasticity [19]. Our review showed that they could be of particular interest to investigate cases of intoxication by alcohol, cocaine, methamphetamines, or opioids, possibly information with sets of CpGs as markers [19,35].

However, many limitations can be already identified. First, other substances frequently influence the epigenetic response to substances of abuse. For instance, in animal models, nicotine was shown to prime the response to cocaine inhibiting histone deacetylase (and thus causing the activation of FosB) in the striatum [48]. Moreover, the effects of substances of abuse like alcohol, cocaine, opiates, and methamphetamine can be also mediated by the concurrent use of antibiotics like ceftriaxone (a third-generation broad-spectrum beta-lactam antibiotic) or butyric acid [49]. Indeed, ceftriaxone increases the transcription of GLT1, thus enhancing the reuptake of the glutamate (an excitatory neurotransmitter) and reducing the drug reward [49]. On the other hand, butyric acid exposure is related to diet and to the molecules produced by aerobic microbial fermentation of polysaccharides in the gut, and may inhibit HDAC, thus reducing the drug reward [49].

Post-mortem use of these markers has its own specific limitations. For example, the tissues used for the analysis cannot be randomly chosen because, even in non-stimulated subjects, different anatomical regions (e.g., NAc and prefrontal cortex) can show different methylation signatures, and even if/when exposed to drugs, the responses tend be more similar [46].

A key limitation is likely to be represented by post-mortem stability of the changes: as shown by our review, epigenetic alterations are usually temporary, and data about the relationship between these changes and post-mortem interval are extremely scarce, although current evidence supports this, at least in the first day post-mortem [36]. Moreover, in many sudden deaths in the young, when attributed to toxic origin, the toxicological history is unknown [50]. This is extremely relevant because, as shown by our review, there can be a difference in the epigenetic changes depending on the pattern of use. For example, in animal models, acute cocaine exposure causes temporary acetylation and phospho-acetylation of histone H4, while repeated cocaine exposure induces persistent H3 acetylation and H3K9 demethylation [51]. Moreover, our review showed that the method of use of the substance is currently underexplored: only one of the reviewed papers was focused on a specific method of use (intravenous injection) [32].

Finally, a significant variable of forensic interest is represented by age. First, because age—as said—influences the epigenome [3,24]. However, age is also relevant to discriminate whether an epigenetic variation is actually “acquired”: epigenetic changes are being considered as one of the explanations of the high heritability of some addictions—like alcohol use disorder—and, for example, epigenetic variations of 65 loci at birth as associated with higher risk of substance abuse during adolescence [52,53,54].

## 5. Conclusions

Current evidence on drug-related epigenetic changes is mainly valid only in animal models. However, solid data obtained from clinical and post-mortem studies on human subjects should be considered as the foundation for forensic research on the use of these changes as toxicological fingerprints as confirmatory tests in highly ambiguous cases. Data should be adjusted for confounding variables like age and (for post-mortem studies) time since death. The possible influence of other substances should be always considered as a possible limitation (and should lead to rigorous anamnesis, in particular aimed at analyzing the use of interfering substances, like nicotine and antibiotics).

## Figures and Tables

**Table 1:** Comparison between epigenetic analysis and conventional toxicological methods in forensic applications.

Feature	Epigenetic Analysis	Conventional Toxicology
Detection window	Can reveal long-term or past exposure (weeks–months, sometimes persistent after cessation)	Detects recent exposure (hours–days); hair analysis can show chronic exposure but limited by availability/condition of hair
Specificity	May identify substance-specific molecular signatures (CpGs, miRNAs)	Identifies parent compounds and metabolites, usually substance-specific
Sensitivity to matrix condition	DNA methylation relatively stable post-mortem; useful when metabolites degraded	Drug metabolites degrade post-mortem; reduced reliability with decomposition
Cost and complexity	High cost; requires specialized equipment and bioinformatics expertise	Lower cost; standardized and widely available methods (GC-MS, LC-MS/MS)
Validation	Limited forensic validation; many findings from small or experimental cohorts	Fully validated and standardized for forensic use
Potential applications	History of abuse, ambiguous cases, post-mortem toxicology, transgenerational/environmental exposure	Routine forensic toxicology (e.g., acute intoxications, impaired driving, doping controls), history of abuse, ambiguous cases, post-mortem toxicology

**Table 2:** Main epigenetic markers associated with substance use in human studies, including target genes, tissue, and type of regulation (M = methylation; R = RNA-based).

Substance	Marker	Reference	Examples of Sites	Target Tissue	Kind of Epigenetic Event
Alcohol	23 CpGs	[12]	SCL7A11, JDP2, GAS5, TRA2B, SLC43A1, PHGDH, PRPF8, ANKS3, TPD52L1, LAMA3, DHX16, DYRK2, SHMT2, SLC1A5, TCF3, RNLS	peripheral blood	M
Alcohol	miR-34	[19]	SIRT1	NAc	R
Cocaine	186 CpGs	[20]	PCDH9	peripheral blood	M
Cocaine	3 miRNAs (miR-124 miR-153 miR-9)	[1]	NFAT5, PLCB1, KCTD20	peripheral blood	R
Methamphetamine	235 CpGs	[26]	TTL7, SCN1A, APBA1, UNC5D, TGFBR3 NET1	frontal cortex	M
Methamphetamine	6 miRNAs (miR-4799, miR-4776, miR-550b, miR-9, miR-181a, miR-15b, miR-let-7e miR-let-7d)	[19]	PPP1CB, MAP2K1, MAPK1	peripheral blood	R
Opioids	1298 CpGs	[30]	JUP, CHKB-CPT1B, ATP11A, SEMA6B	orbitofrontal cortex	M
Opioids	4 long noncoding RNAs	[35]	MEG3, MIAT, NEAT1, NEAT2	nucleus accumbens	R
Cannabis	183 CpGs	[28]	PTGIR, COL18A1, AHRR, CSNK1E	sperm	M

## Data Availability

Data sharing not applicable. No new data were created or analyzed in this study.
